# Two *cis*-regulatory SNPs upstream of *ABCG2* synergistically cause the blue eggshell phenotype in the duck

**DOI:** 10.1371/journal.pgen.1009119

**Published:** 2020-11-13

**Authors:** Li Chen, Xiaorong Gu, Xuetao Huang, Rui Liu, Jinxiu Li, Yiqing Hu, Guoqin Li, Tao Zeng, Yong Tian, Xiaoxiang Hu, Lizhi Lu, Ning Li

**Affiliations:** 1 Zhejiang Academy of Agricultural Sciences, Hangzhou, China; 2 State Key Laboratory for Agrobiotechnology, China Agricultural University, Beijing, China; Uppsala University, SWEDEN

## Abstract

Avian eggshell color is an interesting genetic trait. Here, we report that the blue eggshell color of the domestic duck is caused by two *cis*-regulatory G to A transitions upstream of *ABCG2*, which encodes an efflux transporter. The juxtaposed blue eggshell allele A-A exhibited higher promoter activity and stronger nuclear protein binding capacity than the white eggshell allele G-G. Transcription factor analysis suggested differential binding capability of CTCF between blue eggshell and white eggshell alleles. Knockdown of *CTCF* expression significantly decreased the promoter activity of the blue eggshell but not the white eggshell allele. DNA methylation analysis revealed similar high methylation of the region upstream of the CTCF binding sites in both blue-eggshelled and white-eggshelled ducks. However, DNA methylation levels downstream of the binding sites were decreased and 35% lower in blue-eggshelled ducks than in white-eggshelled ducks. Consistent with the *in vitro* regulatory pattern of causative sites, *ABCG2* exhibited higher expression in uteruses of blue-eggshelled ducks and also showed polarized distribution in their endometrial epithelial cells, distributing at the apical surface of endometrial epithelial cells and with orientation toward the uterine cavity, where the eggshell is pigmented.

In conclusion, our results suggest that two *cis*-regulatory SNPs upstream of *ABCG2* are the causative mutations for blue eggshells in ducks. The blue eggshell variant up-regulated *ABCG2* expression through recruiting CTCF binding, which may function as a barrier element to shield the downstream region from high methylation levels present upstream. *ABCG2* was identified as the only candidate causative gene for blue eggshells; it may function as an efflux transporter of biliverdin to the uterine cavity.

## Author summary

Avian eggshell color is an interesting genetic trait that has been related to numerous interesting biological functions, such as crypsis, mimicry, and protection from ultraviolet radiation. In ducks, blue eggshells are a dominant Mendelian trait. The color is preferred by customers and has become one of the main breeding targets in laying ducks in China. In this study, we identified that duck blue eggshells are likely caused by two *cis*-regulatory variations that synergistically up-regulate *ABCG2* expression in the uterus. *ABCG2* was identified as the only candidate causative gene for blue eggshell; it may function as an efflux transporter of biliverdin to the uterine cavity, where the eggshell is pigmented. Our study provides useful molecular markers for breeding of blue-eggshelled ducks.

## Introduction

Avian eggshell color is very diverse and has gained sustained scientific attention. This attractive trait has been proposed to account for numerous interesting biological functions, such as crypsis, mimicry, and protection from ultraviolet radiation protection [1, 2]. Blue eggshells have been documented in many bird species [2, 3]. Several studies have proposed the color as a positive sexually-selected signal of female quality (such as in terms of antioxidant capacity), which is selected by males for providing more parental care [4, 5, 6, 7, 8]. This bright eggshell color may also reflect qualities of the egg or offspring, as females laying blue eggs might transmit their antioxidants or antibodies to their offspring [9, 10].

In contract with the diversity of eggshell colors, eggshell pigments are monotonous, with only two main pigments accounting for the diverse eggshell colors: biliverdin, which gives a blue-green color, and protoporphyrin IX, which gives a rusty-brown color [11, 12]. A study on the pigments of eggshells from the extinct Dinornithidae proposed that these pigments were ancient in origin and highly conserved [13]. However, the causative genes for eggshell colors are diverse, with even genes for the same eggshell color differing between species. For example, *SLCO1B3* is considered the candidate causative gene for blue eggshells in the chicken, being exclusively expressed in the uteruses of chickens laying blue eggs and a potential contributor in the transportation of biliverdin to the eggshell. However, *SLCO1B3* is not expressed in the uteruses of ducks [14]. Although blue duck eggs are preferred by customers in China, little is known about the genetic mechanism except for its autosomal dominant inheritance, determined by a single gene [15]. In the present study, we show that blue eggshells in ducks are caused by two *cis*-regulatory SNPs that together up-regulate the expression of ATP-binding cassette sub-family G member 2 (*ABCG2*) in the uterus.

## Results

### Identification of positional candidate loci responsible for the blue eggshell phenotype in ducks

**Fig 1:**
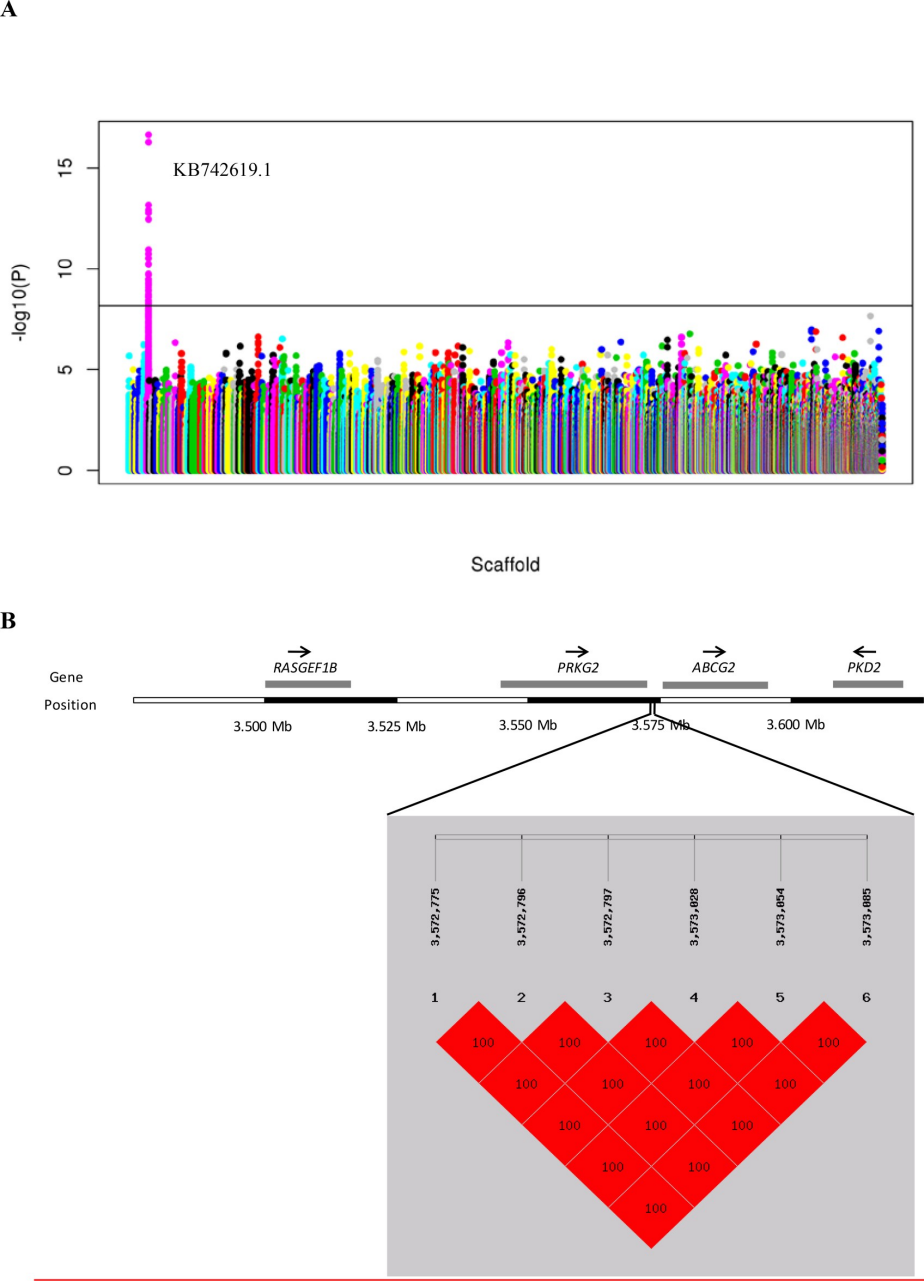
Identification of positional candidate genes for the blue eggshell phenotype in ducks. (A) Manhattan plot of association results from genome-wide association analysis. Y axis shows −log10 (P-value) of association result for each SNP. Each SNP is indicated by a colored dot. SNPs are plotted based on the reference duck genome scaffolds (x-axis). The horizontal solid line is the threshold for the Bonferroni level of significance (p<10−7). (B) Haplotype block at linkage disequilibrium (LD) with the blue eggshell based on six candidate variations. The diagram above the haplotype block indicates genes around candidate variations. Arrows indicate the transcriptional direction.

Positional candidate loci were identified by whole-genome resequencing of 25 blue-eggshelled ducks as well as 24 white-eggshelled ducks. The genotypes of 25 blue-eggshelled ducks were determined with crossing tests, which determined eight birds to be homozygous and 17 heterozygous. In the genome resequencing, an average of eight-fold coverage of the duck genome was generated (**S1 Table**), and variant calling (SNPs and Indels) using these data identified 22,844,207 SNPs and 2,404,821 Indels. Of the identified SNPs, 15,086,311 (MAF >0.05, call rate > = 80%) were used for the genome-wide association study (GWAS). A 283 kb region between nucleotide positions 3,343,841 and 3,627,116 bp in scaffold KB742619.1 showed a significant association with the blue eggshell phenotype (**Fig 1A, S2 Table**). Structural variations and assembly gaps were not found in this region. Genotyping screen of all SNPs and Indels (including 7413 SNPs and 1011 Indels) from this region revealed that six variations displayed perfect genotype-segregation across the three genotypes of resequenced individuals (**S3 Table**). That is, these six variations were fixed for homozygous reference genotype in all resequenced white-eggshelled ducks, fixed for homozygous mutant genotype in all resequenced homozygous blue-eggshelled ducks, and heterozygous in resequenced heterozygous blue-eggshelled ducks. In addition, these six variations showed perfect association with eggshell color in an extended genotyping screen of 1328 additional individuals from a divergent set of breeds/species (**Table 1**). Linkage analysis revealed that they were in complete linkage disequilibrium (LD, r^2^ = 1.0; **Fig 1B**). These results prompted us to speculate that the causative mutation(s) should be among the six variations.

**Table 1:** Genotype segregations of six candidate variations with the eggshell color phenotype in different populations.

Breed/Species	N	Segregations of genotypes with phenotypes (yes/no)								
		Marks	M1	M2		M3		M4	M5	M6
		Position	(3,572,775)	(3,572,796)		(3,572,797)		(3,573,028)	(3,573,054)	(3,573,085)
**Blue-eggshelled ducks**										
Shaoxing duck	1054		1054/0	1054/0		1054/0		1054/0	1054/0	1054/0
Shanma duck	28		28/0	28/0		28/0		28/0	28/0	28/0
Jinyun duck	7		7/0	7/0		7/0		7/0	7/0	7/0
Youxian duck	12		12/0	12/0		12/0		12/0	12/0	12/0
Peking duck	18		18/0	18/0		18/0		18/0	18/0	18/0
Mallard	6		6/0	6/0		6/0		6/0	6/0	6/0
**Total**	**1125**		**1125/0**	**1125/0**		**1125/0**		**1125/0**	**1125/0**	**1125/0**
**White-eggshelled ducks**										
Shaoxing duck	139		139/0		139/0		139/0	139/0	139/0	139/0
Shanma duck	31		31/0		31/0		31/0	31/0	31/0	31/0
Jinyun duck	6		6/0		6/0		6/0	6/0	6/0	6/0
Youxian duck	12		12/0		12/0		12/0	12/0	12/0	12/0
Peking duck	8		8/0		8/0		8/0	8/0	8/0	8/0
Mallard	7		7/0		7/0		7/0	7/0	7/0	7/0
**Total**	**203**		**203/0**		**203/0**		**203/0**	**203/0**	**203/0**	**203/0**

N = number of individuals

### Two *cis*-regulatory SNPs together cause the blue eggshell phenotype

**Fig 2:**
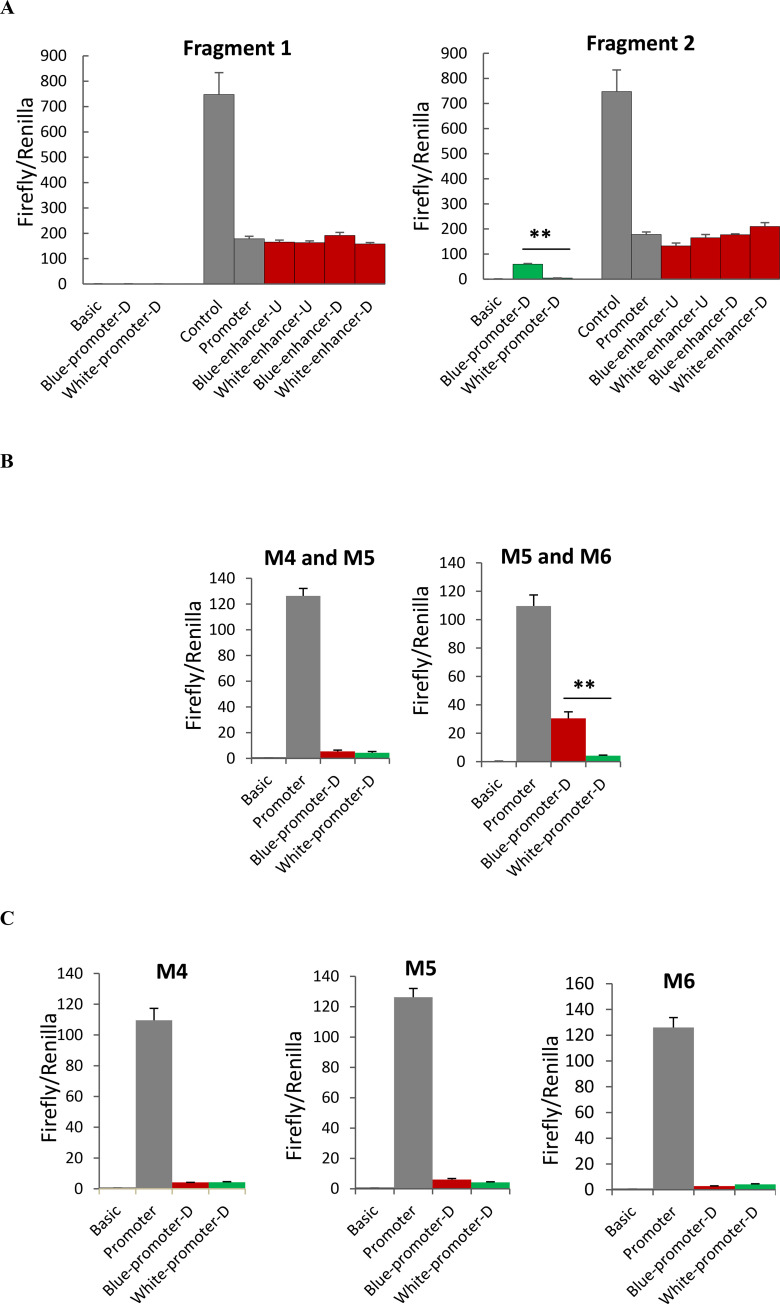
Luciferase assays for the six candidate variations on their enhancer and promoter activity. (A) Two fragments each containing three candidate variations were synthesized: Fragment 1 containing variations M1, M2, and M3, and Fragment 2 containing variations M4, M5, and M6. Each fragment containing the blue eggshell haplotype (Blue-) or white eggshell haplotype (White-) was cloned into the 5’ end of empty pGL3-Promoter and empty pGL3-Basic to analyze their enhancer (enhancer-D) and promoter activity (promoter-D) on the downstream gene, respectively. They were also cloned into the 3’ end of empty pGL3-Promoter to analyze their enhancer activity (enhancer-U) on the upstream gene. Three candidate variations in Fragment 2 were further analyzed for their promoter activity on the downstream gene in pairs (B) or separately (C). Empty pGL3-Basic (Basic) and pGL3-Promoter (Promoter) were used as the reference for promoter and enhancer activity, respectively. Empty pGL3-Control vectors (Control) were used as the positive control for enhancer activity. Data represent the mean±SD from three biological repeats per vector. The activities were compared between the blue and white eggshell vector using Student’s t-test. ** indicates P<0.01.

**Fig 3:**
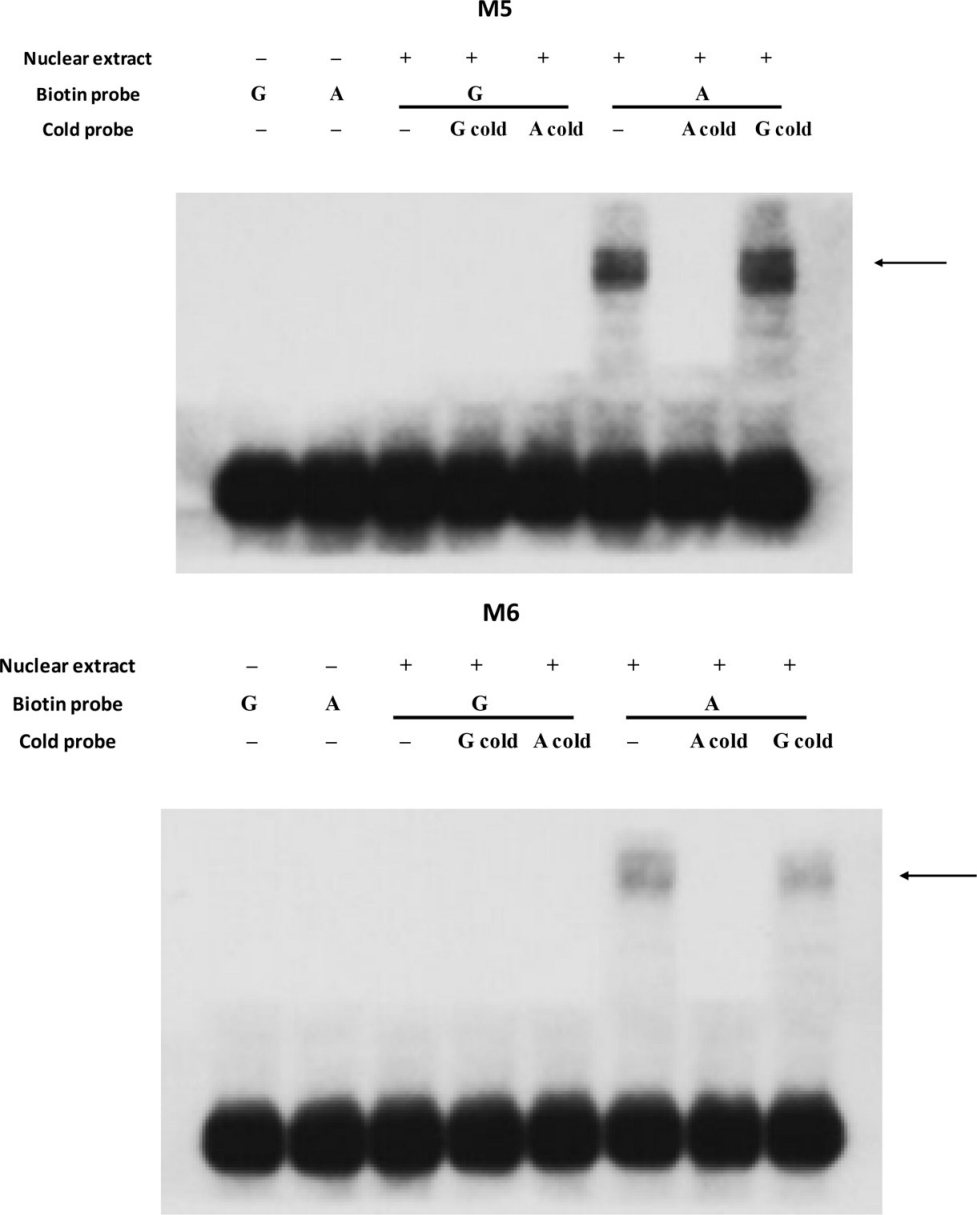
Effects of causative variations on the binding of uterine nuclear proteins. EMSA was performed using biotin-labeled oligo probes containing the blue eggshell allele (A) or white eggshell allele (G). Two microliters of nuclear proteins (5 μg/μl) from uterus were incubated with 40 pmol of biotin-labeled probes. Specific binding was confirmed using 200-fold excess unlabeled cold probes containing the blue eggshell allele (A cold probe) or white eggshell allele (G cold probe). Arrows indicate changes in protein binding capacity between alleles.

Annotation of the duck reference genome suggested that the six candidate variations are located between Protein kinase, cGMP-dependent, type II (*PRKG2*) and *ABCG2*. To determine the exact distance between candidate variations and the two nearby genes, we performed 3’RACE analysis for *PRKG2* and 5’ RACE analysis for *ABCG2* using uteruses from white-eggshelled ducks and blue-eggshelled ducks. We found that neither the transcription end site of *PRKG2* nor the transcription start site of *ABCG2* showed difference between the two genotypes. The six candidate variations were determined to be sited 676 bp downstream from *PRKG2* and 2.6 kb upstream from the transcription start site of *ABCG2* (**Fig 1B**). Considering positions of these candidate variations, we analyzed their enhancer and promoter activity on the downstream gene and enhancer activity on the upstream gene using luciferase assays. For these assays, two fragments each containing three candidate variations were synthesized: Fragment 1 containing variations M1, M2, and M3, and Fragment 2 containing variations M4, M5, and M6. We found that Fragment 1 showed neither enhancer nor promoter activity, while Fragment 2 exhibited promoter activity on the downstream gene (**Fig 2**A). Relative to the promoterless vector (pGL3-Basic), the blue eggshell haplotype of Fragment 2 increased luciferase activity 56-fold, whereas the white eggshell haplotype of Fragment 2 increased the activity only 4-fold, suggesting that while both haplotypes had promoter activity, the blue eggshell haplotype exhibited a much stronger effect than the white eggshell haplotype (**Fig 2**A). Thus, further promoter activity assays were focused on the three candidate variations in Fragment 2, which we assayed separately or in pairs. Results showed that constructs containing the paired variations M5 and M6 exhibited results consistent with those from constructs containing the whole of Fragment 2. Specifically, relative to the pGL3-Basic vector, the blue eggshell construct increased luciferase activity 43-fold, while the white eggshell construct only increased luciferase activity 3-fold (**Fig 2**B); this suggested that the blue eggshell alleles of variations M5 and M6 synergistically increased promoter activity. Although weak promoter activities were also found either for the three separate variations or for the paired variations M4 and M5, in these cases, the blue eggshell and white eggshell alleles showed no difference in luciferase activity (**Fig 2**B and **2**C). Electrophoretic mobility shift assays (EMSAs) further revealed that both variation M5 (SNP, G>A) and variation M6 (SNP, G>A) exhibited differences in nuclear protein binding capacity between blue eggshell and white eggshell alleles (**Fig 3**). These results led us to speculate that variations M5 and M6 are the causative mutations responsible for the blue eggshell phenotype, and that they may induce differential expression of the downstream gene by altering protein binding.

### CTCF binding mediates promoter activity of blue eggshell alleles

**Fig 4:**
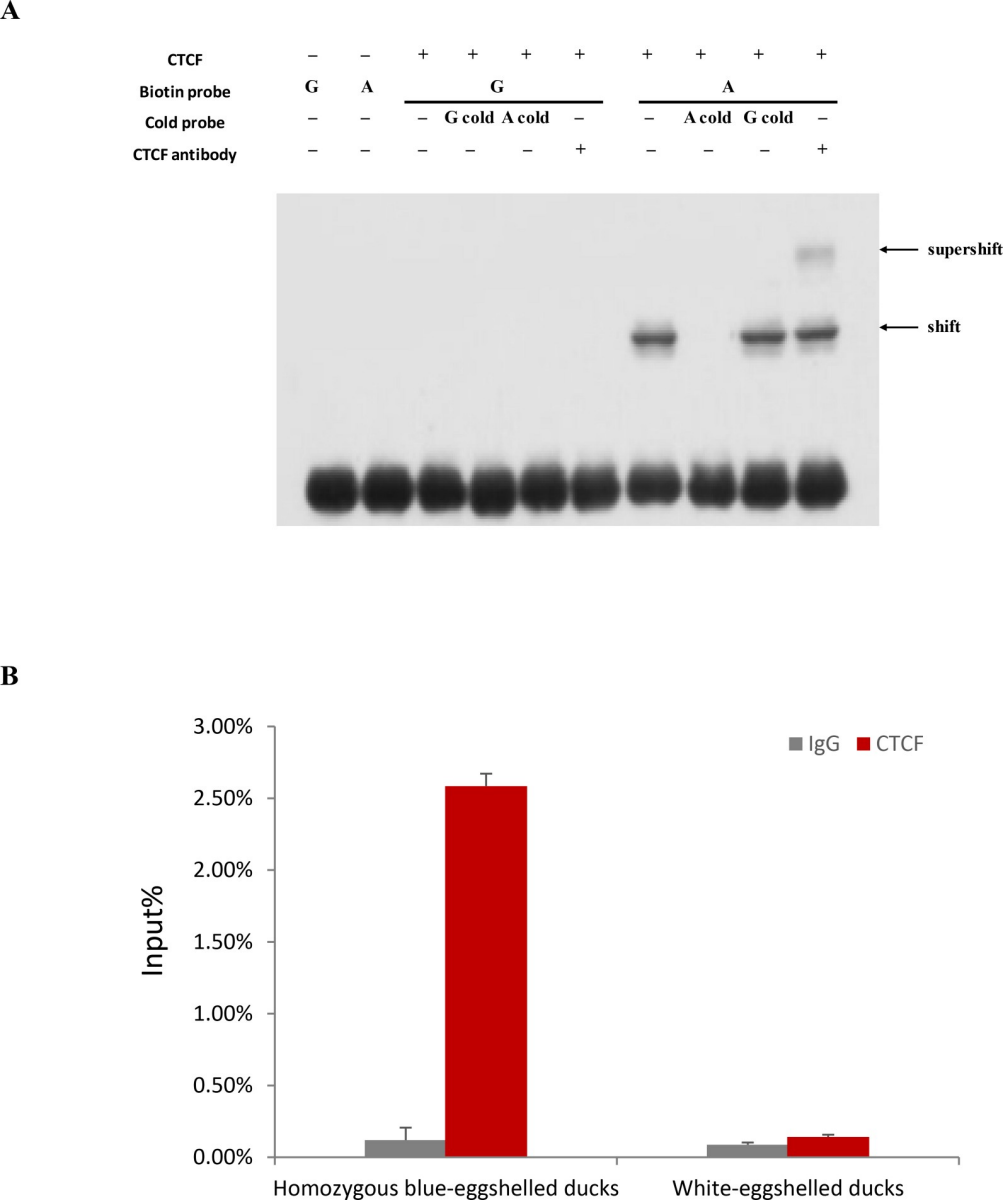
Assay of CTCF binding on variation M5. (A) Supershift EMSA was performed using CTCF antibody. CTCF antibody (1 μg) was incubated with 0.5 μg recombinant human CTCF protein prior to the addition of the binding buffer and probe. G and A represent labelled probes containing the white eggshell and blue eggshell allele, respectively, and G cold and A cold represent cold probes containing the white eggshell and blue eggshell allele, respectively. (B) ChIP-qPCR analysis of CTCF binding on variation M5 in uteruses from blue-eggshelled and white-eggshelled ducks. Chromatin extracts from four individuals of each genotype were immunoprecipitated with CTCF antibody. ChIP-enriched DNA was quantified by qPCR with primers specific for variation M5. Rabbit IgG was used as a negative control. Values of immunoprecipitated samples were normalized to that of the input DNA. Data are presented as mean±SD from four independent individuals.

**Fig 5:**
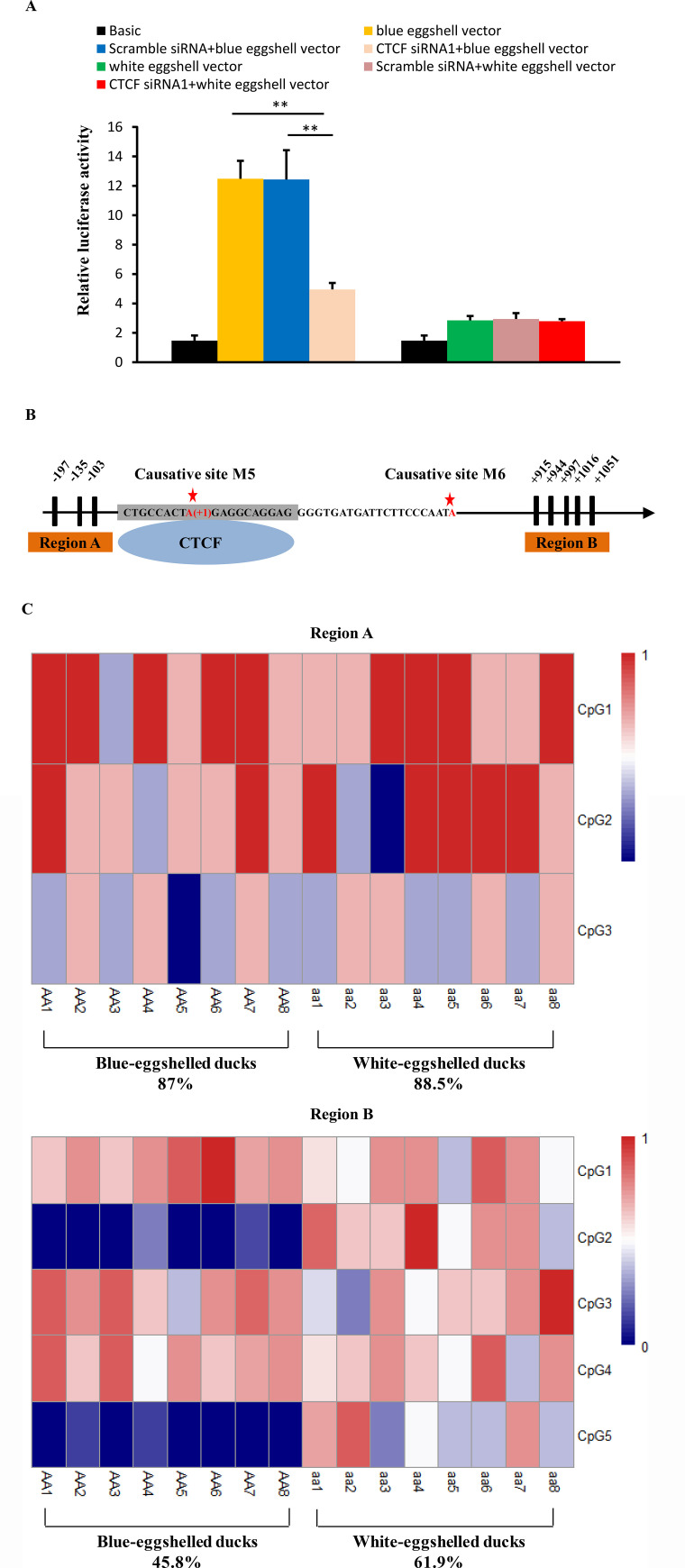
Effects of CTCF binding on the promoter activity of causative sites and methylation status of the region around its binding sites. (A) Effect of knockdown of *CTCF* on the promoter activity of causative sites. The pGL3-Basic vectors containing the blue eggshell or white eggshell alleles inserted in their upstream region were transfected into DEF cells 6 hours after transfection of *CTCF* siRNA1 or negative control siRNA (scramble siRNA) or without siRNA. Cells were collected for luciferase activity analysis 30 hours after siRNA transfection. The group only transfected with the blue eggshell or white eggshell vector was used as control. Data represent the mean±SD from three biological repeats per vector. ** indicates P<0.01. (B) Schematic map of CpGs sites used in the DNA methylation analysis. The causative site M5 was referred as position +1. The black bars represented the CpG dinucleotides upstream or downstream of the predicted CTCF binding sites which were marked in grey background. (C) Heatmap of uterine DNA methylation levels of CpG sites in region A and B of blue-eggshelled and white-eggshelled ducks. The DNA methylation level was determined by sequencing 8 clones of bisulfite treated genomic DNA for each individual. The DNA methylation level of each CpG site was represented by the average methylation of 8 clones. The DNA methylation level from 0 to 1 is shown in the color from blue to red.

To identify proteins exhibiting different binding capacity between blue eggshell and white eggshell alleles, we scanned the surrounding sequences of variations M5 and M6 for predicted transcription factor binding sites. At variation M5, CCCTC-binding factor (CTCF) was predicted to bind to the blue eggshell allele but not the white eggshell allele. To experimentally verify this prediction, we conducted a supershift EMSA using CTCF antibody. We observed that CTCF specifically bound to the blue eggshell but not the white eggshell allele at variation M5, and a supershift was evident upon incubation with CTCF antibody (**Fig 4A**). ChIP-qPCR confirmed this endogenous binding. As shown in **Fig 4B**, CTCF binding fragments were significantly enriched in homozygous blue-eggshelled ducks compared to white-eggshelled ducks. Given that CTCF has been reported to mediate transcriptional activation [16], we hypothesized that CTCF binding might be critical to the promoter activity of the blue eggshell alleles identified in this study. To experimentally verify this hypothesis, we knocked down *CTCF* expression in duck embryonic fibroblast (DEF) cells cotransfected with a luciferase reporter containing either blue eggshell or white eggshell alleles. As expected, reducing *CTCF* expression led to a 2.5-fold loss of luciferase activity of the blue eggshell vector (p = 0.0004), whereas the activity of the white eggshell vector was unaffected (**Fig 5A**), revealing a strong correlation between *CTCF* expression and promoter activation of the blue eggshell alleles.

CTCF can affect DNA methylation [17, 18]. To gain insight into DNA methylation alterations that may accompany CTCF binding, we examined methylation status upstream of the identified binding site, including three CpG sites, as well as downstream, including five CpG sites, in both blue-eggshelled and white-eggshelled ducks (**Fig 5B**). Bisulfite conversion and sequencing analysis of uteruses from eight blue-eggshelled and eight white-eggshelled ducks revealed similar methylation levels between phenotypes in the upstream region, with 88.5% and 87% methylation in white-eggshelled and blue-eggshelled ducks, respectively. However, methylation of the downstream region was 35% lower in blue-eggshelled ducks than in white-eggshelled ducks (**Fig 5C**), leading us to speculate that CTCF binding may decrease the methylation level of the region downstream of its binding site, and it may function as a barrier to shield the downstream gene region from the high methylation level upstream.

### *ABCG2* was identified as the only candidate causative gene responsible for blue eggshell in ducks

**Fig 6:**
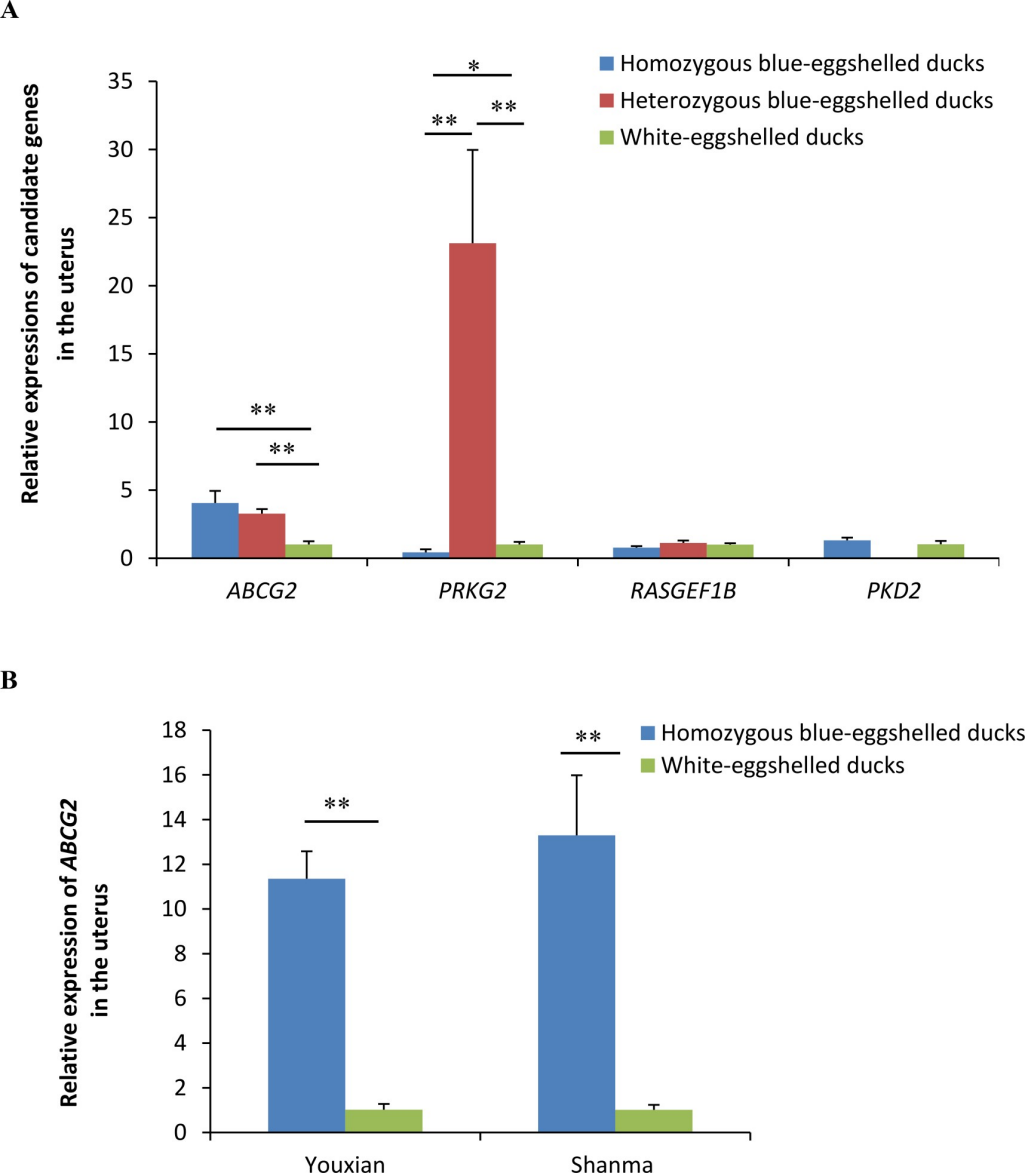
Relative expressions of candidate genes in uteruses from blue-eggshelled and white-eggshelled ducks. (A) Relative expressions of genes adjacent to the causative sites in uteruses from three genotypes of Shaoxing ducks. (B) Relative expressions of *ABCG2* in uteruses from another two duck breeds, Youxian and Shanma ducks. Relative mRNA expression was normalized to *GAPDH* using the 2-ΔΔCt method. Data represent the mean±SD from at least five ducks per group. ** represents p<0.01.

**Fig 7:**
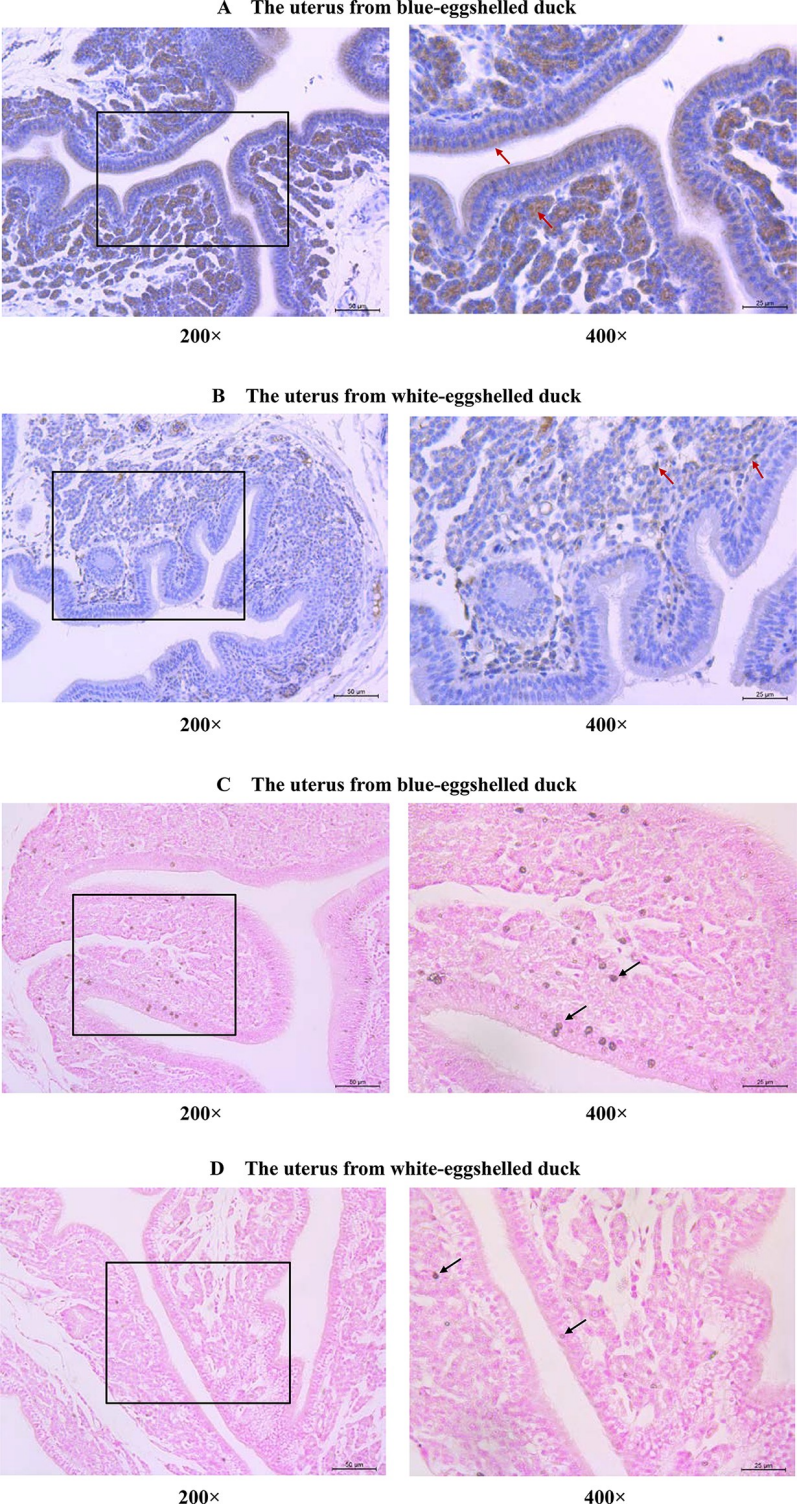
Distributions of ABCG2 and biliverdins in uteruses from blue-eggshelled and white-eggshelled ducks. Immunohistochemical staining of ABCG2 in uteruses from blue-eggshelled ducks (A) and white-eggshelled ducks (B). ABCG2 positive signals were stained in brown (indicated by arrows); the nuclei were stained in blue using hematoxylin. The distributions of biliverdins in uteruses from blue-eggshelled ducks (C) and white-eggshelled ducks (D). The biliverdins were stained in brown (indicated by arrows); the nuclei were stained in red.

We screened candidate genes for the blue eggshell phenotype according to conditions that the candidate gene expression pattern *in vivo* should be consistent with the regulatory pattern of causative sites *in vitro* and should fit the dominant single-gene inheritance pattern. Four genes were found adjacent to the causative sites, with RasGEF domain family member 1B (*RASGEF1B*) and *PRKG2* being upstream and *ABCG2* and Polycystin 2, transient receptor potential cation channel (*PKD2*) being downstream (**Fig 1B**). Expression analysis of these four genes in uteruses from three genotypes of Shaoxing ducks, a Chinese local duck breed, revealed *ABGC2* as the only differentially expressed gene for blue eggshells. Consistent with the regulatory pattern of causative sites, *ABCG2* exhibited significantly higher expression in blue-eggshelled ducks than in white-eggshelled ducks, but no difference between homozygous and heterozygous blue-eggshelled ducks (**Fig 6A**). This phenotype-associated expression was also demonstrated in another two Chinese local duck breeds, Shanma and Youxian ducks (**Fig 6B**). The other three genes were excluded from candidate genes, as they showed similar expression levels between phenotypes, or their expression patterns were inconsistent with the regulatory pattern of causative sites and the dominant single-gene inheritance pattern (**Fig 6A)**. Subsequent immunohistochemical staining of ABCG2 revealed it to be strongly present in the endometrial stromal cells and endometrial epithelial cells of blue-eggshelled ducks; furthermore, its distribution in the endometrial epithelial cells was polarized, being specifically distributed in the apical surface and oriented toward the uterine cavity, where eggshells are pigmented (**Fig 7A)**. However, in white-eggshelled ducks, ABCG2 was only weakly expressed (**Fig 7B)**. Similar to ABCG2, biliverdin, the main pigment of blue eggshells, showed a rich presence in the endometrial stromal cells and endometrial epithelial cells of blue-eggshelled ducks (**Fig 7C)**, while it was almost absent in the endometrial stromal cells and endometrial epithelial cells of white-eggshelled ducks (**Fig 7D)**.

## Discussion

Blue eggshell color is a dominant Mendelian trait in the domestic duck. This bright eggshell color has been considered to have advantages in terms of antioxidant ability and eggshell strength. In this study, our results suggested that two *cis*-regulatory G to A transitions (at positions 3,573,054 and 3,573,085 in scaffold KB742619.1) upstream of *ABCG2* together cause the blue eggshell phenotype. These variant alleles up-regulated *ABCG2* expression through recruiting CTCF, which may function as a barrier element to shield the downstream gene region from high methylation levels upstream. *ABCG2* was identified as the only candidate causative gene for blue eggshell and may function as an exporter of biliverdin.

CTCF is a versatile transcription regulator, with functions relating to gene activation, repression, and chromatin insulation. In our study, CTCF was found to specifically bind to the blue eggshell allele of variation M5. Knockdown of *CTCF* significantly decreased the promoter activity of blue eggshell alleles, suggesting that CTCF is crucial to the promoter effect of blue eggshell variant of M5. CTCF usually influences DNA methylation status around its binding sites [19]. Our DNA methylation analysis showed that methylation levels of the region upstream of the CTCF binding site were similarly high in both blue-eggshelled and white-eggshelled ducks. However, methylation of the region downstream of the binding site was decreased and 35% lower in blue-eggshelled ducks than in white-eggshelled ducks (**Fig 5C**), suggesting that CTCF binding may reduce methylation in the region downstream of its binding site. Acting as a chromatin insulator, CTCF could block the transmission of either active or repressive signals along the chromatin [19]. Based on our findings, we speculate that the blue eggshell variant of M5 leads to CTCF binding, which may function as a barrier element to shield the downstream gene region from high methylation levels upstream, ultimately resulting in higher *ABCG2* expression in blue-eggshelled ducks. We identified that the causative sites were 2.6 kb from *ABCG2*, which position seems like it should locate in an enhancer element rather than a promoter element; however, a clear promoter effect was observed for the causative alleles. We speculate that this may be because of CTCF binding, with CTCF being one of the most important elements in the regulation of chromatin folding [20]. Its binding may promote chromatin folding and bring the causative sites closer to *ABCG2*, thus allowing the causative sites to act as a promoter element.

Biliverdin is the main pigment in blue eggshells. Real-time PCR analysis of two regulatory genes involved in biliverdin biosynthesis, Ferrochelatase (*FECH*) and heme oxygenase-1 (*HMOX1*), revealed similar expression levels between uteruses from blue-eggshelled ducks and those from white-eggshelled ducks (**S1 Fig**); along with our previous transcriptional analysis [21], this suggests there is no difference in biliverdin biosynthesis between the two phenotypic birds. However, biliverdin was found to be differentially distributed between the two phenotypic birds, being richly distributed in the uteruses of blue-eggshelled ducks but weakly distributed, almost absent, in those of white-eggshelled ducks (**Fig 7C and 7D**). We speculate that most biliverdin in the uteruses of white-eggshelled ducks may be reduced to bilirubin, because our previous transcriptome data [21] and a study by Zhang et al. [22] both found that the expression of biliverdin reductase A (*BLVRA*), which can reduce biliverdin to bilirubin, was significantly higher in uteruses from white-eggshelled ducks. The very small amount of biliverdin remaining is not sufficient to pigment the eggshell, thus eventually a white eggshell is formed. Consistent with the abundant biliverdin distribution observed in uteruses of blue-eggshelled ducks, *ABCG2* was highly expressed in uteruses of blue-eggshelled ducks. ABCG2 is an efflux transporter that can transport a wide variety of structurally unrelated compounds. In our study, *ABCG2* was identified as the only candidate causative gene for blue eggshells. We speculate that it may function as an exporter of biliverdin, which is a linear tetrapyrrole. Protoporphyrin IX (PPIX), a cyclic tetrapyrrole, has been demonstrated to be a direct substance transported by ABCG2 [23]. The high expression of *ABCG2* in uteruses of blue-eggshelled ducks could result in abundant biliverdin export to the uterine fluid, pigmenting the eggshell, and eventually a blue eggshell is formed. Consistent with the exporter hypothesis, ABCG2 was found to have a polarized distribution in endometrial epithelial cells: distributed at the apical surface and oriented toward the uterine cavity (**Fig 7A)**, the place where eggshells are pigmented. A transporter function has also been speculated for the chicken blue eggshell gene, *SLCO1B3*; its specific expression in uteruses of blue-eggshelled chickens was speculated to transport biliverdin to the eggshell [14]. Therefore, although the causative gene for blue eggshell differs between chickens and ducks, the functions they play during formation of the blue eggshell color may be consistent.

When analyzing causative gene for the blue eggshell, significantly different expression of *PRKG2* also caught our attention. It showed the highest expression levels in heterozygous blue-eggshelled ducks, followed by white-eggshelled ducks, and the lowest expression levels in homozygous blue-eggshelled ducks. However, we still excluded it from causative gene, as its expression pattern failed to be consistent with the regulatory pattern of causative sites and the dominant single-gene inheritance pattern. Although *PRKG2* is located near the causative sites, and CTCF may affect chromatin state of this region, we speculate that *PRKG2* is beyond the regulatory effect of the identified CTCF binding site. This is because when analyzing methylation status of the region upstream of CTCF binding site, we found that no CpG sites were included in the 3’ end of *PRKG2*. However, we should note that we can’t exclude the possibility that CTCF may regulate *PRKG2* through a different mechanism in its respective promoter.

## Materials and methods

### Ethics statements

Animal care and use were performed according to the regulations in the Guide for the Care and Use of Laboratory Animals issued by the Ministry of Science and Technology of the People’s Republic of China. All animal experiments were approved by the Animal Ethics Committee under the supervision of Zhejiang Academy of Agricultural Sciences (Approval Number: 2018-AEC-2020).

### Animals

In this study, the 25 blue-eggshelled ducks used in whole-genome resequencing were male. Their genotypes were determined with crossing tests in which each male was mated with 20 white-eggshelled females, and the genotype of the male was inferred from the eggshell colors laid by its 20 daughters over a 20-day period during the egg-laying peak. According to the principle that blue eggshell color is a dominant Mendelian trait in ducks, if all 20 daughters laid blue eggs, the male was genotyped as a homozygous blue-eggshelled duck; if about half of the 20 daughters laid blue eggs, the male was genotyped as a heterozygous blue-eggshelled duck. The 24 white-eggshelled ducks used in whole-genome resequencing were female, and were observed laying white eggs for 20 consecutive days during their egg-laying peaks.

An additional 1328 individuals from six duck breeds/species were used for extended genotyping of the six candidate variations. Of the six breeds/species, five are Chinese indigenous breeds originating from Shaoxing, Huzhou, Jinyun, and Wuhan. The wild species, mallard, was captured and then bred in captivity separately by Aoji Duck Farm, which has captured permission granted by the local government. Eggshell colors of all indigenous individuals were recorded over at least 20 days during their egg-laying peaks to determine their phenotypes. Eggshell colors of each mallard were recorded over a reproduction season to determine their phenotypes.

### Whole-genome resequencing

Whole-genome resequencing was performed for 24 white-eggshelled ducks, eight homozygous blue-eggshelled ducks, and 17 heterozygous blue-eggshelled ducks. For each sample, a paired-end sequencing library with an insert size of 400 bp was generated and then sequenced on a HiSeq X-TEN platform according to the manufacturer’s protocol. The resequencing data have been submitted to the SRA database in NCBI with a BioProject accession number PRJNA668048.

### Sequence mapping

Filtered reads were aligned to the duck reference genome (BGI_duck_1.0) using BWA-MEM (version 0.7.12) with default parameters [24]. After alignment, sequencing data in SAM files were sorted according to the reference coordinates, followed by removal of duplicate reads using Picard software package (version 1.107) (http://picard.sourceforge.net). To enhance alignment around Indels, local re-alignments of sequences around Indels were performed with the IndelRealigner tool in the Genome Analysis Toolkit (GATK) (version: 3.8) [25].

### Variation (SNPs and Indels) calling

Variations (SNPs and Indels) were called using the Unified Genotyper implemented in GATK (version: 3.8) and subsequently filtered using the hard filtering process recommended by GATK [25]. For each locus, the genotype with maximum posterior probability was picked as the genotype for that locus.

### Genome-wide association study (GWAS)

GWAS was performed using the Efficient Mixed Model Association eXpedited (EMMAX) method [26]. The matrix of pair-wise genetic distances was used as the variance-covariance matrix for random effect, and the first ten principal components were included as fixed effects.

A Bonferroni corrected *p*-value (P = 1/n, n is the effective number of independent SNPs) < 0.05 was considered to be a genome-wide significant association.

### Genotyping of candidate variations in large populations

An extended genotyping screen for the six candidate variations was conducted in 1328 individuals from six duck breeds. Primers and methods used for genotyping of the six candidate variations are listed in **S4A Table.**

Linkage disequilibrium (LD) for candidate variations (pairwise r2 statistic) was calculated using SHEsis [27] based on the genotyping data from 1328 ducks.

### Luciferase plasmids construction

To analyze promoter and enhancer activity of the six candidate variations by luciferase assay, two 250bp fragments each containing three candidate variations were synthesized: fragment 1 (KB742619.1: 3,572,648–3,572,897 bp) contained variations M1 (at 3,572,775 bp), M2 (at 3,572,796 bp) and M3 (at 3,572,797 bp); fragment 2 (KB742619.1: 3,572,948–3,573,197 bp) contained variations M4 (at 3,573,028 bp), M5 (at 3,573,054 bp) and M6 (at 3,573,085 bp). Each fragment containing either reference or variant alleles was cloned into the pGL3-Promoter vector (Promega, Madison, USA) or pGL3-Basic vector (Promega, Madison, USA) using *NheI* and *XhoI* sites to assay its enhancer or promoter activity on the downstream gene. The fragment was also cloned into the pGL3-Promoter vector using *SaII* and *BamHI* sites to assay its enhancer activity on the upstream gene. To further analyze the promoter activity of the three variations in fragment 2 in pairs or separately, appropriate sequences (3,572,948–3,573,197 bp) were cloned into the pGL3-Basic vector using *NheI* and *XhoI* sites.

### Luciferase assay

DEF cells in 24-well plates were transiently transfected with 720 ng Firefly luciferase vector (pGL3) and 80 ng pRL-TK Renilla luciferase vector (Promega, Madison, USA) using 1.5 uL Lipofectamine 3000 (Thermo Fisher Scientific, Waltham, USA). Twenty-four hours after transfection, luciferase activities were measured with a dual-luciferase reporter assay kit (Promega, Madison, USA) on a SpectraMax M5 (Molecular Devices, Sunnyvale, USA). Renilla luciferase activity was used as the transfection control for normalizing Firefly luciferase activity. Empty pGL3-Basic vector and pGL3-Promoter vector were used as the reference for promoter activity and enhancer activity assays, respectively. Empty pGL3-Control vector (Control) was used as the positive control for enhancer activity. All tests were conducted in triplicate with independent readings in triplicate (n = 3×3 = 9). The data are presented as means with their standard deviation.

### Electrophoretic mobility shift assays (EMSAs) and supershift assay

Uterine nuclear proteins from a blue-eggshelled duck were extracted following the instructions described in the protein extraction kit (TianGen, Dalian, China). Sequences of oligonucleotides used in the EMSA are listed in **S4B Table**. Two microliters of nuclear proteins (5μg/μl) were incubated with 40 pmol of biotin-labeled probes containing blue eggshell allele or white eggshell allele. Simultaneously, cold competition controls were conducted by adding 200-fold excess of unlabeled competitors containing the blue eggshell allele or white eggshell allele. For supershift analysis, the anti-duck CTCF polyclonal antibody made in rabbit using a partial CTCF peptide (**S4C Table**) (HuaAn Biotechnology, Hangzhou, China) was used. CTCF antibody (1μg) was incubated with 0.5 μg recombinant human CTCF protein (Novus biologicals, Littleton, USA) prior to addition of the binding buffer and probe. Complexes were subjected to electrophoresis on non-denaturing 5% polyacrylamide gels. Finally, a picture was obtained using a LightShift Chemiluminescent EMSA kit (Thermo Fisher Scientific, Waltham, USA).

### Chromatin immunoprecipitation (ChIP)-qPCR assay

Briefly, tissues were fixed with 1% formaldehyde, and then chromatin was prepared with a Chromatin Extraction Kit (Abcam, Cambridge, UK). ChIP was conducted with the CTCF antibody using a ChIP kit (Abcam, Cambridge, UK) according to the manufacturer’s instructions. ChIP-enriched DNA was analyzed with qPCR using primers specific for variation M5 (**S4D Table**). ChIP with rabbit immunoglobulin G (IgG) and “input” were used as negative control and positive control, respectively.

### RNA mediated interference for duck *CTCF*

Two sets of siRNAs and one set of negative control siRNA (scramble siRNA) were designed for duck *CTCF*. The siRNA sequences were as follows: siRNA1 5′- GCUCUGAACUGCAUCGUAATT-3′; siRNA2 5′- GCUCUGAACUGCAUCGUAATT-3′; scramble siRNA 5′-UUCUCCGAACGUGUCACGUTT-3′. siRNAs were transfected into DEF cells to analyze their knockdown efficiency using quantitative real-time PCR (qRT-PCR) (**S2 Fig**). siRNA1 exhibited higher knockdown efficiency and was used in subsequent experiments. To assay the influence of *CTCF* expression on the promoter activity of causative sites, 150nM *CTCF* siRNA1 or scramble siRNA was cotransfected with luciferase reporter vectors containing variation sites into DEF cells using Lipofectamine 3000 (Thermo Fisher Scientific, Waltham, USA). Luciferase activities were measured as mentioned above.

### Bisulfite-sequencing

Genomic DNA was isolated by phenol chloroform extraction, after which 2 μg genomic DNA was bisulfite treated with the EZ DNA methylation-Gold kit (ZYMO Research, LosAngeles, USA). To examine the methylation status of targeted genomic regions around the CTCF binding site, we carried out semi-nested PCR with the following conditions. The first round of amplification: 3 min at 94°C; 20 cycles of 94°C for 20 s, 50°C for 30 s and 72°C for 30 s; and a final extension at 72°C for 5 min. The second round of amplification: 3 min at 94°C; 35 cycles of 94°C for 30 s, appropriate Tm for 30 s, and 72°C for 30 s, and a final extension at 72°C for 5 min. Primers used in the semi-nested PCR are listed in **S4E Table.** The anticipated PCR bands were gel-purified using a Gel Extraction Kit (Qiagen, Hilden, Germany), cloned into the pMD19-T Vector (Takara, Dalian, China) and sequenced.

### Rapid amplification of cDNA end (RACE) of *PRKG2* and *ABCG2*

To determine the exact distance between the causative sites and the *PRKG2* and *ABCG2* genes, 3’RACE of *PRKG2* and 5’RACE of *ABCG2* were respectively performed using the 3’ and 5’ RACE System for Rapid Amplification of cDNA Ends kits (Thermo Fisher Scientific, USA). RNA was extracted from duck uteruses using Trizol (TianGen, Dalian, China) and purified using a RNeasy MiniKit (Qiagen, Hilden, Germany). Three micrograms of RNA were used for reverse transcription with SuperScript III (Thermo Fisher Scientific, Waltham, USA) followed by a nested PCR for the first and second amplifications of the 5’and 3’UTRs. Both PCR amplifications were conducted as follows: 95°C for 3 min, followed by 33 cycles of 94°C for 30 sec, 58°C for 30 sec, 72°C for 60 sec, and finally a reconditioning step at 72°C for 7 min. RACE products were purified, sub-cloned into the pMD19-T vector (Takara, Dalian, China), and sequenced. The primers used for RACE are presented in **S4D Table.** The sequences of 3’ and 5’RACE products have been submitted to GenBank with accession numbers MF774072 and MF774071, respectively.

### Relative gene expression analysis

Uteruses were collected from white-eggshelled ducks, homozygous blue-eggshelled ducks, and heterozygous blue-eggshelled ducks. Total RNA was extracted from uteruses with Trizol (TianGen, Dalian, China) and quantified using a Nanodrop2000 (Thermo Fisher Scientific, Waltham, USA). Following cDNA synthesis using M-MLV reverse transcriptase (Promega, Madison, USA), relative gene expression was detected by quantitative real-time PCR conducted on a 7900 Sequence Detection System (Thermo Fisher Scientific, Waltham, USA) under the following conditions: 95°C for 10 min, followed by 40 cycles of 95°C for 30 sec and 60°C for 1 min. The primer sequences used for the real-time PCR are listed in **S4D Table**. Five birds were used for each group. Relative gene expression was normalized to the internal control gene *GAPDH* using the 2^-ΔΔCt^ method [28].

### Immunohistochemistry

In the immunohistochemical assay, the anti-duck ABCG2 monoclonal antibody was made in mouse using a partial ABCG2 peptide (**S4C Table**) (HuaAn Biotechnology, Hangzhou, China).

Fresh uteruses were fixed in 4% paraformaldehyde for 12 hours, processed to make paraffin blocks, and cut into 4 μm sections. The sections were blocked with the blocking solution (8% goat serum and 0.1% Triton-X in PBS) for 30 minutes at room temperature and then incubated overnight at 4°C with ABCG2 antibody (diluted 1:400 in the blocking solution). After washing three times in PBS, the sections were incubated with HRP conjugated goat anti-mouse IgG (1:800, Abcam, Cambridge, UK) for one hour at room temperature and then developed with a DAB Horseradish Peroxidase Color Development Kit (Beyotime, Shanghai, China). The nuclei were counterstained with hematoxylin (HuaAn Biotechnology, Hangzhou, China). Subsequently, the sections were visualized under an inverted microscope at 200× or 400× (Leica, Shanghai, China).

### Biliverdin staining

Paraffin sections were prepared as mentioned above and dewaxed to water under the following conditions: washed with xylene twice for 20 mins, with anhydrous ethanol twice for 10 mins, then with 95%, 90%, 80%, 70% ethanol for 5 mins in turn, and finally with distilled water. Dewaxed sections were stained for biliverdin with Lugo's iodine solution for 6 to 12 hrs and stained for nuclei for 5 mins with nuclear fast red. Subsequently, the sections were visualized under an inverted microscope at 200× or 400× (Leica, Shanghai, China).

## Supporting information

S1 Table Summary of the whole-genome resequencing. (XLSX)

S2 Table Genome-wide association analysis. SNPs with a p-value <10^−7^ are listed. Positions with Bonferroni adjustment < 0.05 were identified as significantly associated and highlighted in boldface.
(XLSX)

S3 Table Genotypes of all SNPs and Indels between positions 3,343,841 and 3,627,116 bp in scaffold KB742619.1. Variations exhibiting perfect genotype segregation among the three genotypes of resequenced samples are highlighted in red.
(XLSX)

S4 Table Sequences used in this study. (DOCX)

S1 Fig Relative expression of *FECH* and *HMOX1* in uteruses from three genotypes of Shaoxing ducks. Relative mRNA expression was normalized to *GAPDH* using the 2^-ΔΔCt^ method. Data represent the mean±SD from at least five ducks per group.
(DOCX)

S2 Fig Knockdown of *CTCF* by siRNA. Two sets of siRNAs were designed for duck *CTCF*. *CTCF* expression was analyzed in DEF cells transfected without siRNA or with siRNAs or negative control siRNA (scramble siRNA). Expression in the without siRNA group (no siRNA) was set as control and normalized as 1, and expression in the siRNA groups is presented as the ratio to that of control. Data represent the mean±SD from three biological repeats per group. ** represents p<0.01.
(DOCX)
