# Supplementary material for: Two cis-regulatory SNPs upstream of ABCG2 synergistically cause the blue eggshell phenotype in the duck
Source: PLoS Genet. 2020 Nov 13;16(11):e1009119. doi: 10.1371/journal.pgen.1009119 (PMC7688135; doi:10.1371/journal.pgen.1009119)
Supplement: S4 Table — (DOCX) [file pgen.1009119.s004.docx]

1. **Primers and methods used in genotyping of the six candidate variations**

| **SNP or Indel marker** | **Primer sequence (5’-3’)** | **Genotyping method** |
| --- | --- | --- |
| KB742619.1: 3,572,775 | AAAAAAAAAAAAAAAAAAAAAAAAAAAAAAAAAAAAAAAAAAAGCTCAGACTGGCAGTTCAGGCTCAA | Snapshot |
| KB742619.1: 3,572,796 | AAAAAAAAAACAAGTACCTCCATGGTGCTGGAGA | Snapshot |
| KB742619.1: 3,572,797 | AAAAAAAAAAAAAAAAAAAGCTGTCCATTCAGATCTCCCAC | Snapshot |
| KB742619.1: 3,573,028 | F: CCTCGGTGCTCTCACTTCTG  R: AGTGGCAGAAGTTAATGG | SSR |
| KB742619.1: 3,573,054 | AAAAAAAAAAAAAAAAAAAAAAAAAAAAAAAAGAATCATCACCCCTCCTGCCTC | Snapshot |
| KB742619.1: 3,573,085 | AAAAAAAAAAAAAAAAAAAAAAAAAAAAAAAAAACCAACTGGTCTTCAACTCAGTCCTC | Snapshot |

1. **Probes used in EMSA and supershift assay**

| **Probe** | **sequence** | **Assayed variation** |
| --- | --- | --- |
| White-M5-F-biotin | 5'-biotin-ACTTCTGCCACTGGAGGCAGGAGGG | 3,573,054 |
| White- M5-F | ACTTCTGCCACTGGAGGCAGGAGGG |  |
| White- M5-R | TGAAGACGGTGACCTCCGTCCTCCC |  |
| Blue- M5-F-biotin | 5'-biotin-ACTTCTGCCACTAGAGGCAGGAGGG |  |
| Blue- M5-F | ACTTCTGCCACTAGAGGCAGGAGGG |  |
| Blue- M5-R | TGAAGACGGTGATCTCCGTCCTCCC |  |
| White-M6-F-biotin | 5'-biotin-ATTCTTCCCAATGGAGGACTGAGTT | 3,573,085 |
| White- M6-F | ATTCTTCCCAATGGAGGACTGAGTT |  |
| White- M6-R | TAAGAAGGGTTACCTCCTGACTCAA |  |
| Blue-M6-F-biotin | 5'-biotin-ATTCTTCCCAATAGAGGACTGAGTT |  |
| Blue- M6-F | ATTCTTCCCAATAGAGGACTGAGTT |  |
| Blue- M6-R | TAAGAAGGGTTATCTCCTGACTCAA |  |

1. **Peptides used for antibody preparation in this study**

| **Experiment** | **Gene** | **immunizing peptide** |
| --- | --- | --- |
| monoclonal antibody preparation | ABCG2 | ESSTNGIPSSKLSPDPADQGGSTLTFHNISYSVKVKSGFLCCRKTASKEVLRDLNGIMRPGLNAILGPTGSGKSSLLDILAARKDPHGLSGDILINGAPQPANFKCTSGYVVQDDVVMGTLTIRENFQFSAALRLPNSVKEQDRNERVNQIIKELGLSKVADSKVGTQFTRGVSGGERKRTNIGMELITDPTILFLDEPTTGLDASTANAVLLLLKRMAKQGKTI |
| polyclonal antibody preparation | CTCF | MEGEAVEAIVEESETFIKGKERKTYQRRREGGQEDDACHI PPNQADGGEVVQDVNSGVQMVMMEQLDPTLLQMKTEVMEGAVPQETEATVDDTQIITLQVVNMEEQPINLGELQLVQVPVPVTVPVATTSVEELQGAYENEVSKGGLQEGEPMICHTLPLPEGFQVVKVGANGEVETLEQGELQPQEDPNWQKDPDYQPPAKKTKKNKKSKLRYTEEGKDVDVSVYDFEEEQQEGLLSEVNAEKVVGNMKPPKPTKIKKKGV |

1. **Primer sequences used in real-time PCR and RACE**

| **Experiment** | **Gene** | **Sequence (5’-3’)** |
| --- | --- | --- |
| **Real-time PCR** | *ABCG2* | F: TACCAAGCATCATCTTCA  R: TAGGACACCATTGTAAGG |
|  | *FECH* | F: TGAACTACTCCAACCCCTACCG  R: TCTTCTTTCCTCTTTGACACAG |
|  | *HMOX1* | F: GCAGAAACTGGAGGGAAGAG  R: CAGATAGCGAGTGTAGGCAT |
|  | *PRKG2* | F: ACCTCTCTATTGGTTGTGCTGG  R: AAGTTCCTTGGTGAGTTCTGCT |
|  | *RASGEF1B* | F: TCACCTTCCTTCTCAGTTCACG  R: CAGGCCAGGCTCACTTAGTCTC |
|  | *PKD2* | F: ATGGGACAGCCTGGACATACAC  R: CCCTGCTCCTTGAGAGGTCTTG |
|  | *GAPDH* | F: TCCAAGGAGTAAGCCAAGCA  R: GGGGAGACAGAAGGGAACAG |
| **ChIP-qPCR** | Binding Site | F: GTCTCTCTGCTTTTTTTCCATTAACTT  R: GCCAACTGGTCTTCAACTCAGTCCTCT |
|  | Control | F: TGGATTCCCTTAGTAATTGGTGAGGAA  R: TGCCACAGCACAACCAGAAGTTCA |
| **3’RACE** | *PRKG2* | PRKG2-3RACE-1F: GATTTCTGATACTTCCTTCTTCCGGGAC  PRKG2-3RACE-2F: AAATTGTGAACCAGAACCACCGAACAGC |
| **5’RACE** | *ABCG2* | ABCG2-311-gsp1: TCCGTTGATCAAAATGTCAC |
|  |  | ABCG2-192-gsp2: TGATGCCATTGAGATCTCTC |
|  |  | ABCG2-129-gsp3: GCTTCACCTTCACACTGTAG |

Note: F and R represent forward and reverse primers, respectively.

1. **Primer sequences used in Bisulfite-sequencing**

| **Region** |  | **Sequence (5’-3’)** |
| --- | --- | --- |
| **Region A** | First round | 1A-1748 GATTTGAATGGATAGTTATGAGTGTGATT  1C-1966 ACCCAAATTACTCAAAACCATAAAATA |
|  | Second round | 1A-1748 GATTTGAATGGATAGTTATGAGTGTGATT  1B-1930 CAAAAAAATATAACAAAAATAAAAACACC |
| **Region B** | First round | 3A-2-2883 TTATTTTGTTAAAGAGAAAATGTTTAGGA  3C-2-3210 ATATCCCCCAACACAAACACC |
|  | Second round | 3B-2-3083 AACTCATAATAAAATCCAACACATTTCTAT  3C-2-3210 ATATCCCCCAACACAAACACC |
