# Supplementary material for: Two cis-regulatory SNPs upstream of ABCG2 synergistically cause the blue eggshell phenotype in the duck
Source: PLoS Genet. 2020 Nov 13;16(11):e1009119. doi: 10.1371/journal.pgen.1009119 (PMC7688135; doi:10.1371/journal.pgen.1009119)
Supplement: S2 Fig — Two sets of siRNAs were designed for duck CTCF. CTCF expression was analyzed in DEF cells transfected without siRNA or with siRNAs or negative control siRNA (scramble siRNA). Expression in the without siRNA group (no siRNA) was set as control and normalized as 1, and expression in the siRNA groups is presented as the ratio to that of control. Data represent the mean±SD from three biological repeats per group. ** represents p<0.01. (DOCX) [file pgen.1009119.s006.docx]

**Fig S2. Knockdown of *CTCF* by siRNA.** Two sets of siRNAs were designed for duck *CTCF*. *CTCF* expression was analyzed in DEF cells transfected without siRNA or with siRNAs or negative control siRNA (scramble siRNA). Expression in the without siRNA group (no siRNA) was set as control and normalized as 1, and expression in the siRNA groups is presented as the ratio to that of control. Data represent the mean±SD from three biological repeats per group. ** represents p<0.01.

**

**
