# Supplementary material for: Two cis-regulatory SNPs upstream of ABCG2 synergistically cause the blue eggshell phenotype in the duck
Source: PLoS Genet. 2020 Nov 13;16(11):e1009119. doi: 10.1371/journal.pgen.1009119 (PMC7688135; doi:10.1371/journal.pgen.1009119)
Supplement: Supplementary file 6 [file pgen.1009119.s005.docx]

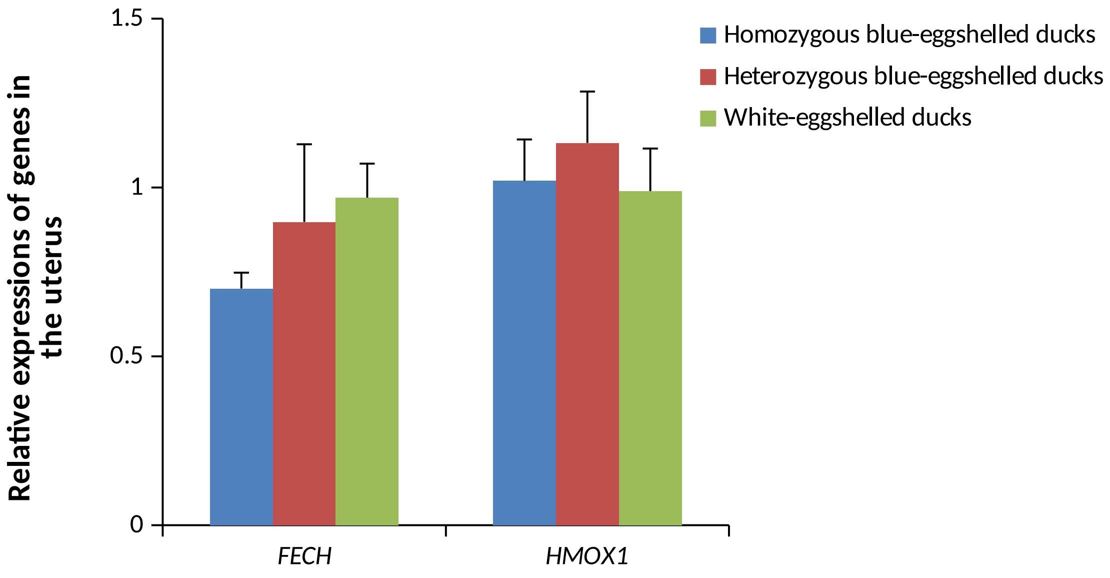
Relative expressions of genes in the uterus
1.5
1
0.5
0
FECH
HMOX1
Homozygous blue-eggshelled ducks
Heterozygous blue-eggshelled ducks
White-eggshelled ducks
